# Reconsidering the Dispositional Essentialist Canon

**DOI:** 10.1007/s11098-021-01607-2

**Published:** 2021-02-03

**Authors:** Samuel Kimpton-Nye

**Affiliations:** University of Bristol, Cotham House, Bristol, BS6 6JL UK

## Abstract

Dispositional Essentialism is a unified anti-Humean account of the metaphysics of low-level physical properties and laws of nature. In this paper, I articulate the view that I label *Canonical Dispositional Essentialism* (CDE), which comprises a structuralist metaphysics of properties and an account of laws as relations in the property structure. I then present an alternative anti-Humean account of properties and laws (still somewhat in the dispositional essentialist spirit). This account rejects CDE’s structuralist metaphysics of properties in favour of a view of properties as qualitative grounds of dispositions and it rejects CDE’s view of laws as relations in favour of a view of laws as features of an efficient description of possible property distributions. I then defend this view over CDE on the grounds that it can overcome an explanatory shortcoming of CDE and that it achieves a level of continuity with science that CDE fails to achieve. The upshot of this paper is a significant narrowing of the range of possibilities in which the *absolutely* best unified account of laws and properties resides.

## Introduction

Scientific theorizing and the discovery of fundamental properties have gone hand in hand. For instance the discovery of the phenomena of electromagnetism and the laws governing them was inseparable from the discovery of the previously unknown, and very likely fundamental, properties of positive and negative charge (Lewis 2009, 205).

There are (at least) three important questions for the metaphysician of science concerning properties and laws of nature:

(i) How should we understand the metaphysics of properties?
(ii) How should we understand the metaphysics of laws of nature?
(iii) How should we understand the relationship between properties and laws?

Since, as Lewis suggests, the formulation of laws has gone hand in hand with the discovery of properties, we might expect an answer to any one of these questions to inform, and be informed by, answers to the others; one might expect to be able to provide a unified account in response the three questions above.

Lewis’s *Humean Supervenience* (HS) is perhaps the most influential such account. According to HS, the world consists of a vast array of properties of, or instantiated at, point-sized regions of space–time (Lewis 1986, ix). Re question (i), properties, according to HS, are *quiddities*; they have no essential features other than primitive self-identity and distinctness from other properties. Crucially, this means that properties do not stand in or induce any necessary connections between distinct existences. Perhaps ours is a world in which everything that instantiates the property F also instantiates the property G. But this does not entail, according to HS, that there is any necessary connection between the properties F and G or between their instances. Re question (ii), laws are features of a particularly efficient *description* of the spatiotemporal distribution of fundamental properties. Laws neither pick out nor induce any necessary connections. And re question (iii), the relationship between laws and properties is that of supervenience—there can be no change in the laws without a change in the spatiotemporal arrangement of quiddistic properties, and (significant) difference in the distribution of properties would be accompanied by a difference in the laws.

Critics have found dissatisfaction with all aspects of HS. First consider *quidditism*, on this view, it is no part of the essence of *charge*, for example, that like charges repel. Like charges repel in this world, but in other worlds charged objects may behave very differently because the true nature of *charge* has nothing to do with the behaviours/dispositions of charged objects that physics discovers. This has led some to object that HS makes properties unknowable in principle and that it is guilty of violating its own naturalistic motivations by positing more structure to the world than physics has use for (see, e.g., Blackburn 1990, 63; Molnar 1999; Bird 2007; Vetter 2015, 8).^1^ On the laws side, it might be objected that laws, according to HS, are not sufficiently modally robust. Since HS laws depend on the thoroughly contingent arrangement of matters of particular fact, there will be cases in which altering matters of fact will alter the laws. As Bird discusses (Bird 2007, 81–86), this leads to some very counterintuitive results because there are situations in which “a law can be removed just by preventing certain interactions” (2007, 84). Relatedly, it has been objected that HS gets the order of explanation between laws and property distributions the wrong way around. One might think that laws should explain the spatiotemporal distribution of properties. However, according to HS, the distribution of properties explains the laws (see, e.g., Armstrong 1983; Bird 2007, 86; Maudlin 2007, 172; Lange 2013).

These problems for HS form a large part of the motivation for Dispositional Essentialism (DE). Dispositional essentialists typically provide very different answers to questions (i)–(iii). Roughly speaking, the dispositional essentialist denies quidditism and maintains that the dispositions associated with properties and discovered by physics are *essential* to those properties. Laws of nature are then understood as flowing from the essences of properties and hence as being necessary (conditional on the existence of the relevant properties). And laws *can* be understood as apt to play a governing role; according to Bird: “Laws […] supervene on potencies […]. Indeed, it is not obvious that this supervenience relation prevents laws from having at least an explanatory function, and, depending on what you think ‘governing’ amounts to, perhaps a governing role also.” Bird argues in some detail (2007, 195–98) against Mumford’s claim that dispositional essentialism precludes laws from playing a governing role. But the main point is that laws, according to DE, are thought to be capable of properly explaining property distributions.

DE is thus advertised as avoiding the epistemological and methodological problems induced by *quidditism*, as rendering the laws sufficiently modal robust and able to explain property distributions, all in an attractively reductive package: at the fundamental level we have just essentially dispositional properties, in terms of which the laws are explained.

More needs to be said, however, about the details of DE. In particular, more needs to be said about what, exactly, it might mean for properties to be *essentially dispositional* and about what it means for laws to *flow from the essences of properties*. In the next section, I’ll outline a particularly influential way of fleshing out these basic ideas behind DE, the view that I call *Canonical Dispositional Essentialism* (CDE). In Sect. 3 I will present an alternative to CDE, which provides its own distinctive set of answers to questions (i)–(iii). In Sects. 4 and 5, I will defend this alternative by arguing, respectively, that it is explanatorily superior to CDE and that it achieves a greater degree of continuity with science than CDE does. What also comes out in Sects. 4 and 5 is an argument that the *particular* unified account of laws and properties that I defend is not merely superior to CDE, but is superior to *any other* combination of the accounts of properties and laws discussed in this paper. There are other accounts of the metaphysics of laws and properties available in the literature that are not explicitly discussed here. So, I cannot claim on the basis of the arguments in this paper to have discerned the *absolutely* best unified account of laws and properties. Nevertheless, what this paper does show is the broad outline of what an absolutely best unified account will look like. While some of the finer details may be open to tweaking, I hope to have significantly narrowed the range of possibilities in which the absolutely best account resides and I hope to focus future attention on developing not just an account of laws or properties in isolation, but the optimal laws-properties unification.

## Canonical Dispositional Essentialism

According to the canonical version of DE (CDE), the essences of fundamental physical properties (*properties* from now on) are exhausted by the modal relations in which they stand to other properties and hence have their identities metaphysically determined by their position in a structure of such relations among properties. Call this *property structuralism*. The canonical view then identifies laws of nature with arcs in the property structure. This version of DE is explicitly endorsed by Chakravartty (2003a; 2007) and is the view towards which Bird (2007) is most inclined. I’ll use this section to articulate these ideas in more detail.

### Property structuralism

The basic dispositional essentialist idea regarding the metaphysics of properties is that properties are *essentially dispositional*. This is often contrasted with quidditism according to which any given property, P, is just essentially self-identical and distinct from other properties and, hence, any connection between properties and dispositions is thoroughly contingent. But what, exactly, does it mean to say that properties are essentially dispositional?

Take the property *fragility* as an example (it doesn’t matter for present purposes that *fragility* is not fundamental, it’s just an easy example to work with). *Fragility* disposes its bearers to *shatter*. The property *shattering* is the manifestation of *fragility*. So, let’s say that the two properties, *fragility* and *shattering* stand in the manifestation relation (M-relation), which is a *modal* relation because a fragile object is disposed to shatter even if in fact it never does shatter. Now, to say that the property *fragility* is essentially dispositional is to say that the M-relation between *fragility* and *shattering* is part of the essence of *fragility*, and, hence, that in any possible world, for any individual x, if x is fragile x will be disposed to manifest shattering. Bird also talks about the stimulus conditions of essentially dispositional properties; *fragility* disposes its bearers to *shatter* when *stressed.* So, the property of being *stressed* is the stimulus of *fragility* and *shattering* its manifestation, on Bird’s view. In which case we might say that the properties *stressed*, *fragility* and *shattering* stand in the stimulus-response (SR) relation. For simplicity in what follows, however, I’ll just talk about the manifestations of dispositional properties, and hence about M-relations instead of SR-relations. But nothing of importance here hinges on this decision and the argument would be unchanged by substituting “M-relations” for “SR-relations” throughout.^2^

Now, according to property structuralism (endorsed by, e.g., Bird (2007), Chakravartty (2003a; 2007) and Mumford (2004)) the essences of *all* properties (all *fundamental* properties, for Bird) are *exhausted* by the M-relations in which they stand to other properties:

[T]he [structuralist] 3 wants the essences and hence identities of her entities to be determined relationally rather than purely intrinsically. (Bird 2007, 139).

A causal property may be identified as the property that it is in virtue of its relations to other properties. (Chakravartty 2003a, 394).

[A property is] nothing more than a set of connections to, and causal powers for, other properties. (Mumford  2004 , 185).

So, *fragility* and *shattering* stand in the M-relation. But these properties will also stand in other M-relations to other properties, which, in turn, stand in further M-relations to *further* properties and so on and so forth. Properties and the M-relations that they enter into, make up a vast network, or *structure* and a property’s essence, and hence identity, is exhausted by the M-relations in which it stands to other properties, according to the property structuralist. In other words, any given property P has its identity fixed by its place in the structure. Properties are *metaphysically* (as opposed to cognitively or epistemically) individuated by their position in the structure of modal relations among properties.

### Laws in CDE

A key motivation for positing essentially dispositional properties is the desire to use those properties to provide a metaphysical explanation of the laws of nature (“One of the nicest features of DIT [the *dispositional identity thesis of properties*] (and an ulterior motive for recommending it) is that it yields an intuitive account of laws” (Chakravartty 2007, 141)).^4^ Dispositional essentialism has been offered in response to the problems faced by *best systems analyses* of laws (e.g., Lewis 1994, 2001) and *nomic necessitation* accounts (e.g., Dretske 1977; Tooley 1977; Armstrong 1999). The former, it has been argued, do not endow the laws with the necessity and metaphysical “oomph” and hence explanatory power that we take them to have, and the latter render the laws mysterious external governing forces.

So, what, exactly, are the laws of nature, according to the structuralist? Both Chakravartty and Bird have expressed the view that laws are relations between properties. The following remarks are representative of Chakravartty’s view:

[N]ot only are laws composed of relations between causal properties; they distinguish and identify properties as well. (2003a, 394).

In a world inhabited by different causal properties, the relations we would there describe as causal laws would be, ipso facto, different as well. (2003a, 402).

Causal laws are never vacuous in principle, given that they are relations between causal properties. (2003a, 405).

Causal laws are nothing more than relations between casual properties. (2007, 150).

In each of these quotes, Chakravartty clearly endorses a view of laws as *relations* between properties.

Now consider the following remarks from Bird, also concerning the nature of laws:

If properties have a dispositional essence then certain relations will hold of necessity between the relevant universals; these relations we may identify with the laws of nature. (Bird 2007, 43).

[L]aws are general relations among properties. (Bird 2007, 200).

[L]aws are general relationships. (Bird 2007, 202).

[L]aws reflect the essential rather than accidental features of potencies and kinds. (Bird 2007, 202).

Since laws flow from the essences of potencies, they must hold in every possible world. (Bird 2007, 5) (where by “potencies” Bird means fundamental, essentially dispositional, properties).

In the first three quotes above, Bird explicitly states that laws are relationships between properties. In the last two quotes, Bird says that the laws flow from the essences of properties. Since, according to Bird’s property structuralism, property essences are exhausted by relations to other properties, we can reasonably interpret the last two quotes as also claiming that laws are relationships between properties.

To be precise, if laws are to be understood as relations, they should be understood as *modal* relations; they have implications for what would or could be the case under certain circumstances. There is no law requiring that a fragile object actually manifests shattering—an object can be fragile and never shatter—but it is plausibly a nomological fact that a fragile object, x, would shatter under the right circumstances. So, a law can relate properties even if one of the properties related is not *actually* instantiated. (See Chakravartty (2007, chap. 5) for more on this in connection with the dispositional essentialist treatment of *vacuous* laws).

M-relations, that is, *arcs* in the property structuralist’s structure,^5^ are modal relations among properties, which (exhaustively) *constitute* the *essences* of those properties. Hence, it seems fair to attribute to Chakravartty and to Bird, both of whom explicitly endorse a structuralist metaphysics of properties and the idea that laws flow from the essences of properties, the view that M-relations *just are* laws. Chakravartty adds further plausibility to this interpretation of CDE when he says: “If the identity of a property is determined by the relations of which it is capable, as described by law statements, then citing these law statements is a necessary precondition for picking out or identifying properties.” (2007, 134).

Earlier I said that there is work to be done in order to precisify the core dispositional essentialist tenets that properties are essentially dispositional and that laws flow from the essences of properties. The *canonical* precisification of these ideas results from combining a structuralist metaphysics of properties, in the sense discussed in Sect. 2.1., with a view of laws as identical with the M-relations that make up the property structure, in the sense discussed here in Sect. 2.2. Canonical Dispositional Essentialism (CDE) earns its *canonical* status, I suggest, because of the prominence of its main proponents (Bird and Chakravartty) in the development of DE in general and because it seems to be the most thorough fleshing out of the dispositional essentialist ideas about the metaphysics of laws and properties and the relation in which properties and laws stand to each other.

## Reconsidering the Canon

I will now present an alternative set of answers, still somewhat in the dispositional essentialist spirit, to questions (i)–(iii) about the metaphysics of properties, laws of nature and the relationship between the two. I’ll call this alternative unified answer to (i)–(iii) *qualitative dispositional essentialism* (QDE).^6^ Then, in Sect. 4, I’ll motivate QDE by showing that it can overcome an explanatory deficiency for CDE before, in Sect. 5, arguing that QDE is continuous with science in a way that CDE is not.

### QDE properties—the grounding view

In answer to question (i), “how should we understand the metaphysics of properties?” QDE pursues a middle ground between the quidditism of HS and the structuralism of CDE. With CDE and against HS, QDE posits necessary connections between properties and dispositions, so, for example, according to QDE, there is no possible world in which like charges are not disposed to repel each other. But with HS and against CDE, QDE denies that the essences of properties are constituted by the dispositional relations in which they stand. QDE denies property structuralism and maintains that properties are self-individuating *qualities*, where qualities are not metaphysically dependent upon a structure or upon any relations in which they may stand.

In order to account for the different behaviours/dispositions associated with different properties, QDE properties ought not be understood as ‘bare quiddities’ where there is nothing more to say about what distinguishes different bare quiddities than that they are numerically distinct. Rather, properties should be understood as ‘thick’ or ‘qualitative’ quiddities (see Hildebrand (2016)), where different qualitative quiddities are distinguished by their different qualitative natures, and the fact that different properties are associated with different dispositions is thus accounted for in terms of their different qualitative natures (see Tugby (2020), Hildebrand (2016), Smith (2016)). In practice, it may be difficult to say anything more about the qualitative differences between low-level properties, such as *charge* and *mass*, than that they are there and account for the different dispositions conferred by the different properties. But this view of properties is nonetheless justified on the basis of the explanatory work it is capable of (more on this in Sect. 4).

In order to secure the necessary connections between properties and dispositions, e.g., between *charge* and the disposition to exert a repulsive force on like charges, QDE maintains that properties fully ground dispositions, so, following Tugby (2020), I will call this view of properties *the grounding view*. Coupled with the plausible idea that grounding is necessitating in the sense that if X fully grounds Y, then it is metaphysically necessary that Y exists if X exists (prominent defenders of *grounding necessitarianism* include Fine (2015), Dasgupta (2014) and Trogdon (2013)), this yields necessary connections between properties and the dispositions that they fully ground. We may still envisage the very same structure of manifestation relations as that envisaged in CDE. But whereas, in CDE, the structure metaphysically individuates and hence grounds properties, in QDE, properties are self-individuating grounds of the structure.^7^

Smith (2016) identifies two kinds of quidditism: I-quidditism and R-quidditism. This distinction helps to shed further light on the grounding view of QDE and its standing in relation to the quidditism of HS and the structuralism of CDE. According to I-quidditism, properties are individuated independently of their theoretical roles or any dispositions/causal powers with which they may be associated (see Smith 2016, 239). And according to R-quidditism, “there are no restrictions on the recombination of properties in metaphysically possible worlds” (Smith 2016, 240). The present suggestion, that properties are qualities that fully ground and hence are necessarily connected with dispositions, is inconsistent with R-quidditism but is consistent with I-quidditism. HS is consistent with both I-quidditism and R-quidditism and CDE is inconsistent with both I-quidditism and R-quidditism.

### QDE Laws—the BSA

In answer to question (ii) about the metaphysics of laws, one could combine the QDE account of properties with a view of laws as arcs in the structure of relations that those properties ground. To take this route would be to stick as close as possible to the CDE conception of laws. However, for reasons that I shall discuss in Sect. 5, QDE, as I define it, includes something far closer to the HS account of laws. Roughly speaking, then, QDE results from combining a view of properties as qualitative grounds of dispositions with a view of laws as features of a description of *possible* spatiotemporal distributions of property instances. I’ll elaborate briefly.

HS, as defended by Lewis, comprises the *best systems analysis* of the laws of nature (BSA). According to the BSA, laws are the axioms of a deductive systematization of all information about the spatiotemporal locations of properties. Strength and simplicity of such a systematization trade off: a very strong system could just list the spatiotemporal location of every property instance. But this would be incredibly complicated and unwieldy. A simpler system might provide fewer, more general statements about the spatiotemporal distribution of properties, from which we could deduce additional information not explicitly given. The optimal system is one that maximises the virtues of strength and simplicity.^8^ The laws, according to the BSA, are the axioms of this optimal system (see, e.g., Lewis (1973; 1983)).

QDE includes an account of laws very much like the BSA of HS. However, instead of just systematizing the spatiotemporal distributions of properties at the actual world, laws, according to the QDE-BSA, are axioms of the strongest, simplest systematization of actual and metaphysically *possible* property distributions (see, Demarest (2017) and Kimpton-Nye (2017) for more detailed articulations of this idea).

The motivation for systematizing metaphysically possible property distributions as well as the actual distribution of properties is roughly as follows. QDE shares with CDE the desire to imbue the laws of nature with necessity so that it may overcome the problems for HS that arise given a view of the laws as thoroughly contingent. By systematizing all metaphysically *possible* distributions of properties, the laws are rendered metaphysically necessary. There are some options regarding *alien* properties^9^: (i) laws may be indexed to sets of world that share properties (Kimpton-Nye (2017) defends this on epistemological grounds), (ii) alien properties may be deemed metaphysically impossible (there may be good naturalistic motivations for this), or (iii) laws may concern worlds that include alien properties, but these would be laws we cannot know about. On any of these options, laws come out necessary because they concern the space of *possible* property distributions, which is itself necessary assuming that what is possible is necessarily possibly as per S5 modal logic. But I would be inclined to recommend option (i) or (ii) to ensure the epistemic accessibility of laws.

QDE also shares with CDE the desire to provide a *reductive* account of natural laws in terms of modally “oomphy” physical properties. Properties, according to QDE, are metaphysically necessarily connected with certain dispositions because they fully ground those dispositions (given the plausible assumption that grounds necessitate what they fully ground). On the plausible assumption that the modality with which dispositions are associated is metaphysical possibility (see, e.g., Vetter (2015) for an extensive and compelling defence of this idea), QDE properties can thus be understood as metaphysically determining various possibilities and, in particular, as determining the full range of metaphysically *possible property distributions*. It follows that the *best systematization* of all possible property distributions is, in turn, metaphysically determined by the properties. So, given the QDE-BSA, the laws of nature are necessary and are metaphysically explained by the modally *oomphy* properties, in keeping with the spirit of dispositional essentialism. In the remainder of this paper I will defend the QDE laws-properties package just articulated.

## In Defence of QDE—explanation

In response to a question about why opium puts patients to sleep, Bachelerius, a prospective doctor of medicine in Molière’s *Le Malade Imaginaire*, says that “there is a dormitive virtue in it, whose nature it is to make the senses drowsy.” This answer, given by Bachelerius in his *viva voce* examination, is received enthusiastically by his examiners, much to the amusement of the audience.

Bachelerius’ humorously bad explanation seems to turn on paraphrasing the explanandum—*opium’s ability to put patients to sleep*—as *dormitive virtue*, which is then said to be part of the nature of opium. But it does not suffice as an explanation of *why* opium puts people to sleep to be told that it is in the *nature* of opium to put people to sleep. The purported explanation appears to just tell us what we already know, albeit phrased slightly differently.

CDE succumbs to a similar explanatory pitfall—laws/M-relations are said to constitute the essences of properties, and properties are said to explain the laws of nature. So, for example, in response to the question “why do charged objects interact in accordance with Coulomb’s law?”, the CDE answer would be (roughly) “because Coulomb’s law is part of the essence of the property *charge*”. But an explanation of laws in terms of properties according to which it is part of the essence of properties that those laws hold seems as dissatisfying as Bachelerius’ “explanation” of opium’s ability to make a patient sleep in terms of its *dormitive virtue*.

In this section, I’ll precisify this worry with an argument to the conclusion that CDE laws and properties symmetrically ground each other, which makes the explanation of laws in terms of properties circular. I’ll explain how QDE does better by breaking this symmetry and providing a properly *reductive* picture. I’ll also argue (in 4.2) that DE’s explanatory aspirations cannot be satisfied *just* by identifying laws with regularities (the point holds whether regularities are understood as things in the world or descriptions) as opposed to M-relations, i.e., problems remain for the combination of a structuralist view of properties and a BSA account of laws.

###  Symmetrically grounded laws and properties in CDE

As discussed in Sect. 2.1, CDE posits a structure of M-relations. One question that we may ask is what (if anything) grounds this structure? Yates (2018) talks about properties, on the structuralist conception, as *composing* a structure:

[Structuralism]^10^ require[s] that entities can be individuated by their places in a structure composed by the entities themselves. (Yates 2018, 4543).

In [structuralism], powers are fully individuated by their places in a type-casual structure fully composed of powers. (Yates 2018, 4544).

Granting that properties compose the structure, and that composition is a grounding relation, it follows that *properties* ground the structure.

However, one might object to the idea that properties ground structure given that the option remains to posit the structure as an ungrounded primitive entity. The main precedent for this idea is ontic structural realism (OSR). According to OSR, *individuals* are eliminated from the ontology in favour of an ontologically primitive relational structure (e.g., French and Ladyman 2003; Ladyman et al. 2007). *Prima facie* talk about individuals is then understood as merely abstracting from the fundamental relational structure. Since, on this view, structure is all there is, it certainly is not the case that the structure ontologically depends on anything else. Perhaps, then, the structuralist about properties could say something similar, namely, that a relational structure is all there is to properties, in which case it would be false to say that this structure ontologically depends on properties, or anything else. However, this interpretation of structuralism about properties shares with OSR a commitment to relations without relata, which is something that many have found objectionable (e.g., Chakravartty 1998, 2003b; Psillos 2006a); according to Chakravartty “one cannot intelligibly subscribe to the reality of relations unless one is also committed to the fact that some things are related” (1998, 399). In the interest of avoiding such controversies, it would seem reasonable to follow Yates’ understanding of structure as ontologically dependent upon the properties that “compose” it—CDE properties ground the structure.

Recall that, according to property structuralism, the structure, S, at a world, *w*, *grounds* the properties at *w* because it metaphysically individuates them. Hence, properties and structure symmetrically ground each other.^11^ Odd as this may sound, things get worse for CDE because, as I shall now show, it follows that properties and *laws* symmetrically ground each other, which directly threatens CDE’s aspirations to (asymmetrically) explain laws in terms of properties.

In Sect. 2.2, it was argued that CDE laws are identified with M-relations. Now, if you fix the M-relations (that is the *laws*, according to CDE) at a world, *w*, you fix the property structure at *w*. If we let [M] denote the plurality of M-relations/laws at a world, *w*, and let S denote the property structure at *w*, then we may ask the following question: what is the relationship between [M] and S? Answer: *identity*. The plurality of M-relations at a world, *w*, *just is* the structure at *w*.

Now, if the collection of M-relations, [M], at a world, *w*, and the property structure, S, at *w* are identical, then anything true of S must be true of [M] too. According to property structuralism, the structure, S, at *w* grounds the properties at *w* because it metaphysically individuates them. But if S grounds the properties at *w* then [M] must ground properties at *w* too, since S and [M] are identical. But M-relations, I have argued, just are the laws, according to CDE. Thus, the collection of M-relations at *w*, [M], just is a collection of laws. To say that [M] grounds properties, then, is just to say that *laws* ground properties, given CDE. To be clear, the argument runs as follows:

(i) S = [M].
(ii) [M] is a collection of laws (from the CDE account of laws).
(iii) S grounds properties(from the CDE account of properties).
(iv) [M] grounds properties (from i, iii and Leibniz’s Law).
(v) Laws ground properties (from ii and iv).

The conclusion that laws ground properties is antagonistic to CDE’s aspiration to explain laws in terms of properties. Grounding is plausibly understood as an explanatory relation distinct from, but analogous to *causation* (Fine 2012, Sect. 1), see also Schaffer (2016); just as one may explain some fact A by saying that some temporally prior fact B *caused* it, one may explain A in terms of B by saying that B *grounds* A. An explanation of A in terms of B will not do, however, if in order to explain B, we must appeal to A—this kind of *circularity* of explanation is vicious. The type of explanation of the laws that the dispositional essentialist should seek to provide is a grounding explanation whereby properties ground laws. But if laws ground properties too, then the explanation is circular and thus fails to satisfy a key motivation for dispositional essentialism, which was to explain laws in terms of properties, not vice versa.^12^

Another way of seeing the problem is as follows: according to CDE, M-relations constitute the essences of properties, and M-relations are laws. Structuralist DE thus builds laws into the essences of properties and then seeks to *explain* the laws of nature in terms of those very property essences (c.f. Jaag (2014)). But *this* looks a lot like explaining the fact that opium induces sleep in terms of opium’s dormitive nature.

A further problem highlighted by the above argument is that CDE multiplies the number of *fundamental* entities. Dispositional essentialism, at least on the face of it, promised an attractively reductive picture; at the fundamental level we just have physical properties which *ground* the laws of nature. What the above argument shows, however, is that according to CDE, properties cannot be more fundamental than laws because each grounds the other. The picture presented, then, is one of properties and laws as equifundamental, which is a far less parsimonious picture than one according to which it is just properties at the fundamental level.

QDE breaks the symmetry by denying that properties are in any way ontologically dependent upon the structure of M-relations. Properties, according to QDE, are self-individuating qualities that asymmetrically ground a structure of M-relations. And laws, according to QDE, are axioms of the best deductive systematization of all actual and possible property distributions. QDE is thus preferable to CDE in two significant respects: it avoids symmetrically grounded properties and laws and hence avoids any Molière-type concerns, it also avoids positing *both* properties and laws at the fundamental level.

### Laws as regularities?

Perhaps the obvious response to the symmetrical grounding problem for CDE is to maintain that laws are universal generalizations, or *regularities*, of the form ∀x(Fx → Gx), as opposed to M-relations, viz*.*, arcs in the property structure. Indeed, Bird himself considers this option^13^:

According to the regularity version of dispositional essentialism about laws, laws are those regularities whose truth is guaranteed by the essentially dispositional nature of […] properties. (2007, 46–47, my emphasis).

The idea, then, is that M-relations do the work of *guaranteeing the truth of*, or *grounding* (depending on whether regularities are understood as linguistic entities or entities in the world) and hence explaining, nomic regularities. But surely no M-relation is *identical* with any regularity, in which case premise ii. in the symmetrical grounding argument of the previous subsection can be rejected and the circularity concern is blocked. While properties cannot explain laws *qua* M-relations, properties can explain laws *qua* regularities, or so one might think.

But how, exactly, are we to understand the claim that properties explain regularities? According to an argument from Barker and Smart (2012), the property structuralist component of CDE cannot achieve even this.

According to the property structuralist, there is nothing more to the essence of a property than the M-relations in which it stands to other properties—all properties have their essences *exhausted* by M-relations. Hence, all that the property structuralist may appeal to in answering the question about how properties, F and G, say, explain the regularity ∀x(Fx → Gx) are the M*-*relations in which F and G stand to other properties (Tugby 2012, 725, highlights this nicely). To clarify the point, it will help to introduce some abbreviations:

M(F, G) = the fact that the M-relation holds between F and G.

R = the fact that if x is F then x is disposed to manifest G.

We can thus understand the property structuralist as positing a necessary connection between M(F, G) and R to explain the regularity ∀x(Fx → Gx). It is no *accident*, according to the property structuralist, that if M(F, G) holds then R holds, hence it is no accident that if M(F, G) holds then every x that is F will be G too. Thus, the regularity, ∀x(Fx → Gx), is explained by M(F, G)’s holding.

But now we may ask: what accounts for the necessary connection between M(F, G) and R? Why is it that in every possible world in which M(F, G) holds, every x that is F will be G? Perhaps there is some *third*-order relation, M*, between the relational facts M(F, G) and R, which ensures that if M(F, G) holds then R holds too. We can denote the situation like this: M*[M(F, G), R]. But now we may ask what accounts for the connection between M*[M(F, G), R], M(F, G) and R? What ensures that if M*[M(F, G), R] holds at a world, w, then if M(F, G) holds R holds too? Is there some *fourth-*order relational fact, M**, such that M**[M*[M(F, G), R], M(F, G), R]? We are clearly off on a regress of higher and higher order M-relations. (This is the nub of Barker and Smart’s “ultimate argument against dispositional monist accounts of laws” (2012), see also Barker (2013).^14^)

Perhaps the regress can be blocked by repudiating the initial explanatory demand. The property structuralist might say that there is a brute necessary connection between the fact that the M-relation holds between F and G and the fact that for all x, if x is F then x is disposed to manifest G. That is to say, there is a brute necessary connection between M(F, G) and R such that in any world in which M(F, G) holds, R holds too. By maintaining that the necessary connection between M(F, G) and R is just brute, the property structuralist needn’t appeal to higher order relations and the regress is blocked.

However, appeal to brute necessary connections is antagonistic to the motivation for property structuralism, and CDE more generally, which was to explain patterns of property distributions, not in terms of some brute necessary connections, but in terms of powerful properties. As Tugby puts it, “One of the main intuitions behind dispositionalism is that the properties of things are not inert: they pack a powerful punch; they give a causal ‘biff’ to their possessors.” (2012, 726). But if it turns out that all the work is being done by brute necessary connections between higher-order relational facts, then this motivation for CDE seems not to have been satisfied.

The point, then, is that it will not help one with anti-Humean/dispositional essentialist sympathies, who is concerned by the symmetrical grounding problem, to identify the laws with regularities instead of M-relations. Furthermore, it doesn’t matter if the regularities in question are more sophisticated, as per the BSA. As mentioned in Sect. 3.2, the dispositional-BSA account of laws (Demarest 2017; Kimpton-Nye 2017) still wants the laws/regularities to be metaphysically explained by properties, which it achieves by maintaining that properties metaphysically explain their distributions which, in turn, metaphysically explain the BSA-laws. But what the Barker-Smart problem shows is that structuralism about properties precludes those properties from explaining regularities in property distributions, and if properties cannot metaphysically explain their own distributions, they cannot explain the BSA laws. Whether laws are M-relations, simple regularities or sophisticated BSA-regularities, a structuralist metaphysics of properties is not cut out to do the explanatory work that dispositional essentialism requires. We are thus pushed towards more radically rethinking dispositional essentialism and QDE constitutes just such a radical rethink.^15^

### An opacity worry for QDE

The opacity worry for QDE is a challenge to add plausibility to the claim that *qualities* ground dispositions. As Tugby puts it “…opponents […] might object […] that the alleged internal connection between qualities and dispositions is itself pretty opaque.” (2012, 729).^16^

Schaffer defines a relevant sense of “opacity” as follows:

*Opacity*: It is opaque as to why the obtaining of the ground state is linked to the obtaining of the grounded state if and only if the proposition that the ground state obtains without the grounded state obtaining is [conceivable/logically possible/a priori open]. (Schaffer 2017, 4).

The worry, then, is that the purported grounding link between quality and disposition is opaque in the above sense—it is certainly conceivable, logically possible and a priori open that a quality, positive charge, say, exists in the absence of a disposition to exert an attractive force on instances of negative charge. So why think that any such grounding link exists?

Schaffer has argued, however, that a purported grounding link’s being opaque is not a good reason to reject that link out of hand. This is because Schaffer advocates an abductive methodology in metaphysics according to which metaphysical posits are justified to the extent that they are explanatorily fruitful (Schaffer forthcoming, pp. 8–9) (see also (Lewis and Lewis 1970; Paul 2012 for ideas relating to and in defence of this abductive methodology in metaphysics)).

It may be explanatorily fruitful, and hence justified, to posit the existence of a grounding link even if that link is not *transparent* in the sense that its existence is evident to pure reason or a priori knowable. For example, and assuming that sets exist, Schaffer suggests that we are justified in positing a grounding link (namely, set formation) between Socrates and {Socrates}. This is because, given that Socrates exists and that the set formation grounding link exists between Socrates and {Socrates}, it is no accident that {Socrates} exists given the existence of Socrates, so the link is explanatorily fruitful:

To buttress this […] claim [that a grounding link obtains between Socrates and {Socrates}], note that the theoretical role of explanation includes (i) revealing patterns, (ii) providing recipes, and (iii) allowing understanding [Schaffer 2018 ]. Seeing {Socrates} as the output of Socrates via set formation (i) reveals a unifying pattern that extends through Plato and {Plato}, Aristotle and {Aristotle}, etc.; (ii) conveys a recipe to wiggle the existence of {Socrates} by wiggling the existence of Socrates; and (iii) allows one to understand why {Socrates} exists, by revealing how sets are collected. (Schaffer forthcoming, 2).

Schaffer discusses other examples from mereology (2017, forthcoming), meta-ethics and quantum ontology (forthcoming). In each of these examples, we are justified in positing a grounding link between two entities because doing so is explanatorily fruitful in the sense that it reveals patterns, provides a recipe, and allows understanding. What’s more, the grounding link in each case is opaque in the above sense that it is conceivable/logically possible/a priori open that the ground state obtained without the obtaining of the grounded state. For example, it is conceivable that Socrates existed without the existence of {Socrates} and it is conceivable that some wood arranged table-wise existed without the existence of a table (mereological nihilism is conceivable). Nevertheless, we are justified in positing grounding links in these cases—set formation in the former and a mereological fusion principle in the latter—because doing so is explanatorily fruitful.

So, according to Schaffer, the existence of grounding links need not be evident to pure reason or knowable a priori, i.e., they need not be *transparent*. Rather, we can take a more holistic, abductive approach to metaphysics, according to which we are defeasibly justified in positing grounding links if they are explanatory. In this sense, Schaffer thinks that there is an important analogy between grounding and causation:

Those who would demand a priori grounding principles strike me as akin to those in the early days of the sciences who demanded a priori causal principles (“rational mechanics”). We have come to recognize the need for substantive dependence functions in concrete causal cases. I am trying to extend this insight to concrete metaphysical cases. (Schaffer 2017, 14).

According to this Schafferian line, then, it is no objection to QDE that the grounding link between quality and disposition is opaque. Indeed, we have defeasible justification for believing in grounding links between qualities and dispositions given their explanatory fruitfulness. And the point of the discussion thus far has been that QDE, “opaque” grounding links and all, *is* explanatorily fruitful. QDE is explanatorily superior to Humean accounts of laws and properties because it guarantees that the laws are sufficiently modally robust (see sect. 1), and it is explanatorily superior to CDE because it breaks the problematic symmetry and equifundamentality between laws and properties.

Schaffer’s argument for the explanatoriness of the set formation grounding link (see second Schaffer quote above) can be mirrored for QDE: positing a grounding link between positive charge and a disposition to exert an attractive force on negatively charged individuals (i) reveals a unifying pattern that extends through mass and a disposition to exert an attractive force on massive individuals, etc.; (ii) conveys a recipe to wiggle the existence of a disposition by wiggling the existence of a quality; and (iii) allows one to understand why certain dispositions exist by revealing how they depend on the existence of certain qualities.

QDE’s “opaque” grounding links are explanatorily fruitful—this has been the main thrust of the argument of the present section. We are thus justified in believing in the existence of these grounding links given a holistic, abductive approach to metaphysics.

Perhaps one could object that the analogy between Socrates grounding {Socrates} and qualities grounding dispositions breaks down because whereas we have independent reason to believe in the existence of Socrates, the only reason for positing qualities is to ground dispositions and laws.^17^ Admittedly, qualities, in the present sense, are more “theoretical” than individuals such as Socrates—the main reason for positing qualities is the theoretical work that they can do and the theoretical work of concern here is explaining dispositions and laws. But qualities can also do other theoretical work besides accounting for dispositions and laws, such as explaining objective similarity and difference. Indeed, accounting for similarity and difference is generally a role assigned to natural properties, so one could understand the present discussion as a case in favour of understanding the metaphysics of natural properties in such a way that they can also explain laws and dispositions.

## In Defence of QDE—continuity with science

For all that’s been said, one could opt for an account of properties as self-individuating, qualitative grounds of dispositions, as per QDE, but instead of adopting a BSA-type account of laws, one could stick closer to CDE and maintain that laws are arcs in the dispositional structure that properties asymmetrically ground. In this section, I defend the BSA-laws component of QDE.

The following is a plausible desideratum on a philosophical theory of laws:

CONTINUITY: an account of laws should be continuous with actual scientific practice.

Scientists are largely concerned with discovering laws of nature and philosophical interest in laws is due to their central importance to science. So, a philosophical account of laws ought to yield something close to the important scientific concept, and something that scientists could reasonably hope to have epistemic access to. A philosophical account of laws that is not continuous with science in this way risks irrelevance.

Lewis derives the idea of balancing the virtues of strength and simplicity, which is central to the BSA account of laws, from actual scientific practice:

I take a suitable system to be one that has the virtues we aspire to in our own theory-building, and that has them to the greatest extent possible given the way the world is… [I]t must be as simple in axiomatisation as it can be without sacrificing too much information content; and it must have as much information content as it can have without sacrificing too much simplicity. A law is any regularity that earns inclusion in the ideal system. (Lewis 1983, 367).

The standards of simplicity, of strength, and of balance between them are to be those that guide us in assessing the credibility of rival hypotheses as to what the laws are. (Lewis 1986 , 123).

Scientists use strength and simplicity as epistemic guides in the *discovery* of laws; the strongest, simplest statements about how the world is are assumed most likely to be the laws of nature. The QDE-BSA (like the original BSA) takes the scientists’ epistemic principles and makes them constitutive of laws. In this way, the QDE-BSA achieves continuity with actual scientific practice by sticking close to the scientific understanding of laws and by ensuring the epistemic accessibility of the laws via scientific methods, which is good at *discovering* strong, simple statements about the world. As Dorst puts it: “it is as if Lewis asked himself: ‘How should one design a theory of laws to make sense of the ways that scientists investigate them?’. And his response was this: ‘Figure out what sorts of things scientists’ epistemic practices would be good at discovering, and make those things the laws’.” (Dorst 2017, 880). (See also Hall (2015)).

M-relations, in contrast with QDE-BSA laws, are highly specific—they are individual arcs in a potentially huge structure. Thus, taken individually, M-relations would likely not be very informative. Listing *all* the M-relations would be very informative, but it would be far from simple; indeed, a list of all the M-relations would likely be far too complex and unwieldy to be of any use at all to intellectually limited creatures like us.

Furthermore, epistemic accessibility of specific M-relations is far from guaranteed. As mentioned, science appears to be in the (successful) business of formulating very general statements about the world. But the contribution made by any given M-relation to the most general goings on in the world is likely to be intimately tied up with its interactions with a whole host of other M-relations. It is not obvious that we should be able to easily isolate and (epistemically) identify particular M-relations independently of the whole tangled web of M-relations which *as a whole* is responsible for the regularities we observe in nature.

Identifying laws with all and only the M-relations thus has the significant disadvantage of failing to respect CONTINUITY; M-relations don’t look much like the scientific conception of laws as strong simple statements about the world and they risk being epistemically inaccessible via scientific methods of inquiry. (See Kimpton-Nye (2017) for a detailed discussion of how to ensure the epistemic accessibility of laws if something akin to the BSA is combined with an unHumean account of properties). To ensure the generality and epistemic accessibility of laws, we thus seem compelled to *zoom out* to the larger scale patterns in possible property distributions and to identify those patterns with laws; this is exactly what the QDE-BSA recommends.

The following is a further plausible desideratum on a theory of laws.

PRAGMATISM: part of what it is to be a law of nature is to afford predictive utility to creatures like us.

PRAGMATISM is closely related to CONTINUITY; the continuity with science that the QDE-BSA achieves ensures that the laws have the predictive utility that PRAGMATISM demands. A strong systematization will carry lots of information, including information about the future and about other possibilities, which is what we need to make predictions. And a simple systematization will be easy for cognitively limited being such as ourselves to wield in order to actually make those predictions (see Dorst (2017) on this point, though Dorst argues that the “nomic formula” we will need to include principles in addition to strength and simplicity if BSA laws are to properly satisfy the pragmatic demand).

Conversely, failure to respect CONTINUITY, as in the case in which laws are identified with M-relations, compromises PRAGMATISM for three reasons: particular M-relations are highly specific and so will be of limited predictive power, the list of all M-relations will likely be very long and complicated and so of limited use to *us* in making predictions, there is no guarantee that all or even many of the M-relations that there are will be epistemically accessible to us, so even if M-relations were highly predictive in theory, we may not be able to tap into this predictive power.

To summarize this section: the QDE-BSA is preferable to an account of laws as identical with M-relations because the former, but not the latter, is continuous with actual scientific practice, which implies that the former, but not the latter, yields laws that are useful for creatures like us.

## Conclusion

This paper discussed two different anti-Humean accounts of the metaphysics of properties: structuralism and the grounding view. And it discussed two different accounts of the metaphysics of laws: laws-as-M-relations and the BSA. CDE resulted from the combination of structuralism and laws-as-M-relations and QDE resulted from the combination of the grounding view and the BSA. I argued that QDE is superior to CDE because it avoids Molière-type explanatory concerns for CDE and it achieves a greater degree of continuity with actual scientific practice than CDE does. I also argued that the remaining two ways of packaging together some combination of the above accounts of properties and laws are inferior to QDE. Combining structuralism with the BSA does nothing to address Barker and Smart’s explanatory regress (Sect. 4.2) and combining the grounding view with laws-as-M-relations does not achieve the same level of continuity with science as is achieved by the QDE combination of the grounding view with the BSA. The desiderata of explanatoriness and continuity with science thus give us a good sense of what the *absolutely* best laws-properties package deal will look like. While there may be some wiggle room on the precise details of the metaphysics of laws and properties, properties should not have their essences *constituted* by anything that they are then expected to explain, this was the lesson of the symmetrical grounding argument and of Barker and Smart’s “ultimate” argument. And laws should be the sort of things that are useful to creatures like us in our practical and scientific endeavours, this was the lesson of the continuity with science argument.

## Footnotes

1 Lewis’s stated motivation for Humean Supervenience was to “resist philosophical arguments that there are more things in heaven and earth than physics has dreamt of” (Lewis 1994, 474).

2 It is worth noting, however, that Vetter (2015, chap. 2) has argued forcefully against understanding dispositional properties in terms of their stimulus and manifestation conditions, suggesting instead that dispositional properties be understood in terms of manifestations alone. Relatedly, Vetter defends the idea that the modality with which dispositions are most fundamentally associated is metaphysical possibility. I follow Vetter in this respect.

3 Bird calls his structuralist view of properties *dispositional monism*.

4 This does not apply to Mumford, who argues that essentially dispositional properties obviate the need for laws.

5 M-relations will likely be *directed*; *shattering* is the manifestation of *fragility*, and not vice versa.

6 The name is borrowed from Tugby (2012). Tugby uses “QDE” to refer just to an account of the metaphysics of properties that he argues is able to overcome a problem raised by Barker and Smart (2012) against property structuralism. I also endorse this account of properties and for similar reasons to those discussed by Tugby. But in this paper, I will use “QDE” more broadly to refer to the combination of the aforementioned account of properties and a BSA-type account of laws.

7 This account of properties is just the view of properties that Tugby (2012) defends in response to an explanatory problem for property structuralism (see Barker and Smart 2012; Barker 2013) – more on this later.

8 For simplicity, I omit discussion of *fit*.

9 Thanks to an anonymous referee for encouraging me to mention this issue.

10 Yates actually uses the term “PPO” here, for “pure powers ontology”, but the idea is the same – that all fundamental properties are essentially and exhaustively dispositional and so have their identities fixed by the relations in which they stand to other properties, viz. place in a structure.

11 This is consonant with more moderate versions of OSR. Esfeld and Lam (2008), for example, maintain that objects and relations are on an ontological par “with objects being characterized only by the relations in which they stand” (Esfeld and Lam 2008, 27).

12 Perhaps there are other cases in which symmetrical grounding is kosher. The point here is just that symmetrically grounded laws and properties would be antagonistic to the motivations for DE.

13 Though Bird ultimately leans more heavily towards the view of laws as relations as evidenced by the official definition of laws later in his book: “The laws of a domain are the fundamental, general explanatory *relationships* between kinds, quantities, and qualities of that domain, that supervene upon the essential natures of those things.” (Bird 2007, 201, my emphasis).

14 The regress is modified to fit my set up in terms of M-relations. Barker and Smart stick with the standard conception of dispositions in terms of stimulus and manifestation conditions, which they call SR-relations. Hence the regress they articulate is of higher and higher order SR-relations.

15 One might be concerned that Barker and Smart’s “Ultimate Problem” trumps the symmetrical grounding problem of Sect. 4.1. However, the Ultimate Problem specifically targets the property structuralist’s claim to be able to explain *regularities* in the universe in terms of structuralist properties, and isn’t directly a problem just for the view according to which *laws* are identical with arcs in a property structuralist structure, which is the target of the symmetrical grounding problem. So, the two problems affect slightly different aspects/variants of CDE.

16 See also Barker (2013) and Sider (2020, chap. 2) for a similar challenge.

17 Thanks to a reviewer for raising this point.
